# Loop technique for guidewire manipulation during endoscopic ultrasound‐guided hepaticogastrostomy

**DOI:** 10.1002/jgh3.12903

**Published:** 2023-04-12

**Authors:** Haruo Miwa, Kazuya Sugimori, Yuto Matsuoka, Kazuki Endo, Ritsuko Oishi, Masaki Nishimura, Yuichiro Tozuka, Takashi Kaneko, Kazushi Numata, Shin Maeda

**Affiliations:** ^1^ Gastroenterological Center Yokohama City University Medical Center Yokohama Japan; ^2^ Division of Gastroenterology Yokohama City University Graduate School of Medicine Yokohama Japan

**Keywords:** biliary drainage, EUS‐guided hepaticogastrostomy, guidewire manipulation, interventional EUS, loop technique

## Abstract

**Background and Aim:**

Endoscopic ultrasound‐guided hepaticogastrostomy (EUS‐HGS) is widely used in the management of biliary obstructions; however, literature on guidewire manipulation is lacking. This study aimed to assess the utility and optimal conditions of the loop technique for guidewire manipulation during EUS‐HGS.

**Methods:**

Consecutive patients who underwent EUS‐HGS between April 2015 and January 2022 were included in this study. Patient characteristics and procedural details were retrospectively analyzed. Guidewire manipulations were classified as conventional technique or loop technique, based on the shape of the guidewire tip.

**Results:**

A total of 52 patients (Median age: 73 years, 38 male and 14 female) underwent EUS‐HGS. The median guidewire insertion time was 49 s and the median overall procedure time was 20.5 min. The initial guidewire direction was toward the peripheral side in 23 patients (44%). Technical success rate of the EUS‐HGS was 100%. Twenty patients (38%) underwent the procedure using the loop technique and 32 (62%) with the conventional technique. In the logistic regression analysis, an angle between the bile duct and needle of >70° was independently associated with use of the loop technique (OR 9.84; 95% CI: 2.24–43.13; *P* <0.01).

**Conclusion:**

This study revealed the utility of the loop technique in EUS‐HGS. This technique is recommended if the bile duct is punctured at an angle >70°.

## Graphical abstract

This study evaluated the utility of the loop technique in EUS‐HGS and the optimal conditions for its use. The loop technique is recommended if the angle between the bile duct and needle is >70° on fluoroscopic images.

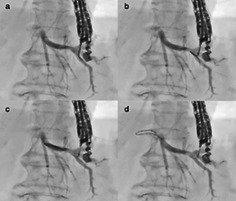

## Introduction

Endoscopic ultrasound‐guided hepaticogastrostomy (EUS‐HGS) is widely used in the treatment of patients with biliary obstructions in whom the transpapillary approach has failed.^1^, ^2^, ^3^ Indications for EUS‐HGS include duodenal obstruction, altered anatomy due to prior surgery, difficulty in cannulation, and hilar biliary obstruction.^4^, ^5^, ^6^

EUS‐HGS involves several steps such as puncture, guidewire manipulation, tract dilation, and stenting.^7^, ^8^ To date, many procedures and devices have been reported to facilitate a successful EUS‐HGS^9^, ^10^, ^11^, ^12^, ^13^, ^14^; however, there are few published reports pertaining to guidewire manipulation during the procedure.^15^, ^16^ Nevertheless, adverse events associated with guidewire manipulation can occur because no dedicated guidewire for use in EUS‐guided procedures has been developed so far.

Conventionally, guidewire manipulation in EUS‐HGS is performed by rotating the angled tip to advance it into the hilar side of the bile duct. However, excessive rotation or frequent pushing and pulling of the guidewire may result in unwanted consequences, such as guidewire shearing.^17^ The loop technique using an inverted guidewire tip is useful in difficult cases; however, there are no reports analyzing the use of this technique in multiple procedures. The present study aimed to assess the utility of, and optimal conditions for, the loop technique of guidewire manipulation in EUS‐HGS.

## Methods

### Patients

Consecutive patients who underwent EUS‐HGS for the treatment of biliary obstruction between April 2015 and January 2022 were included in this study. Written informed consent was obtained from all patients prior to the procedure. The exclusion criteria were as follows: patients who refused consent, whose movies of the procedure were not saved, and in whom anything except the 0.025‐in. guidewire was used. The study protocol was approved by the institutional review board of Yokohama City University (approval number: F220300060), and all procedures conformed to the provisions of the Declaration of Helsinki (as revised in Fortaleza, Brazil, October 2013).

### EUS‐HGS procedure

EUS‐HGS was performed using a convex echoendoscope (UCT‐260; Olympus, Tokyo, Japan) and an ultrasound system (UE‐ME2 premier, Olympus). The intrahepatic bile duct in the lateral hepatic lobe, bile duct of segment 3 (B3), or segment 2 (B2) was punctured with a 19‐G fine needle aspiration (FNA) needle (EZ shot 3, Olympus). After injection of the contrst medium, the flat‐panel detector of the fluoroscopy system (Ultimax‐i, Canon Medical Systems, Tochigi, Japan) was rotated so that the punctured bile duct was shown in the long axis. Subsequently, a 0.025‐in. guidewire (VisiGlide 2, Olympus) was inserted. If contrast injection or guidewire insertion failed, the FNA needle was withdrawn and the other branch was punctured again. Once the guidewire advanced beyond the left hepatic duct, the needle was removed and tract dilation was performed using a mechanical dilator, balloon dilator, or electrocautery dilator. Finally, plastic stents or self‐expandable metallic stents were deployed, as per the requirement of each patient's disease or condition.

### Definition of guidewire technique

**Figure 1:**
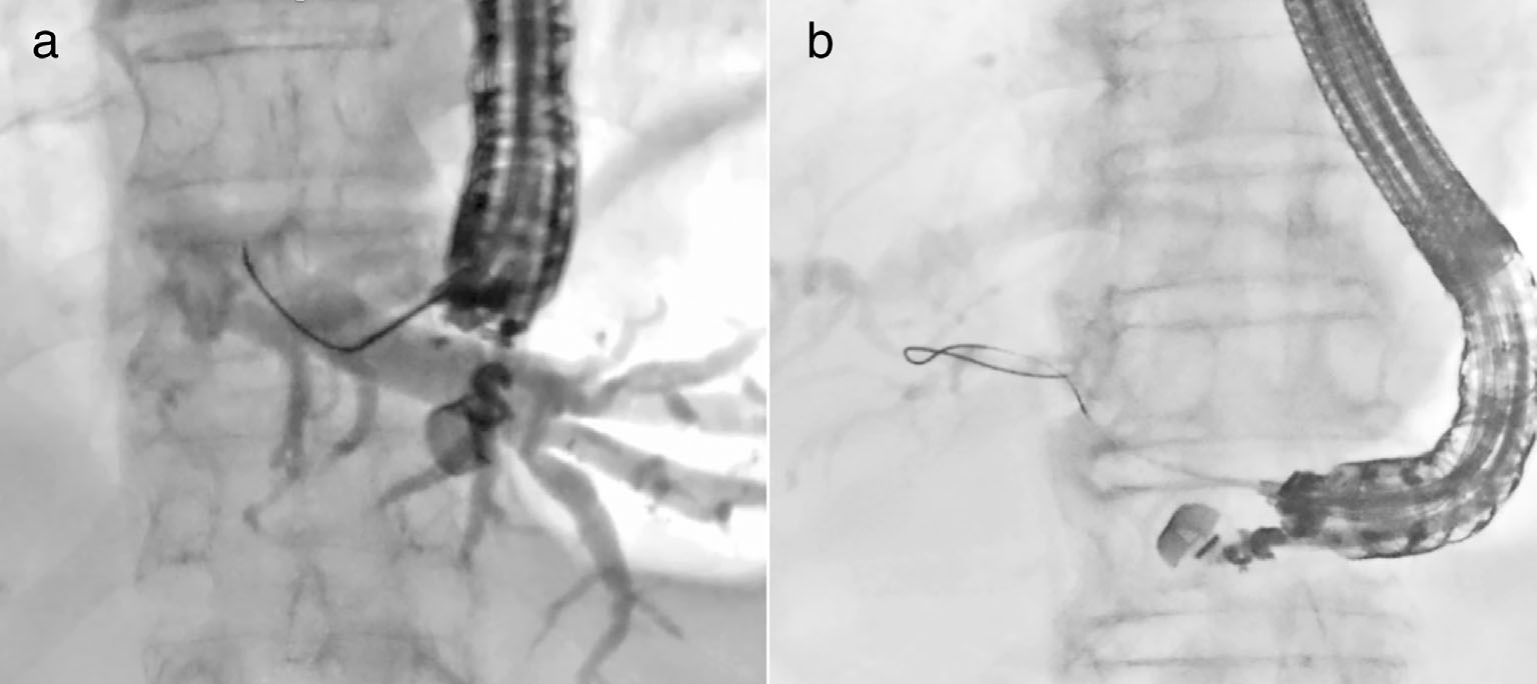
Guidewire manipulation techniques. (a) Conventional technique. (b) Loop technique.

**Figure 2:**
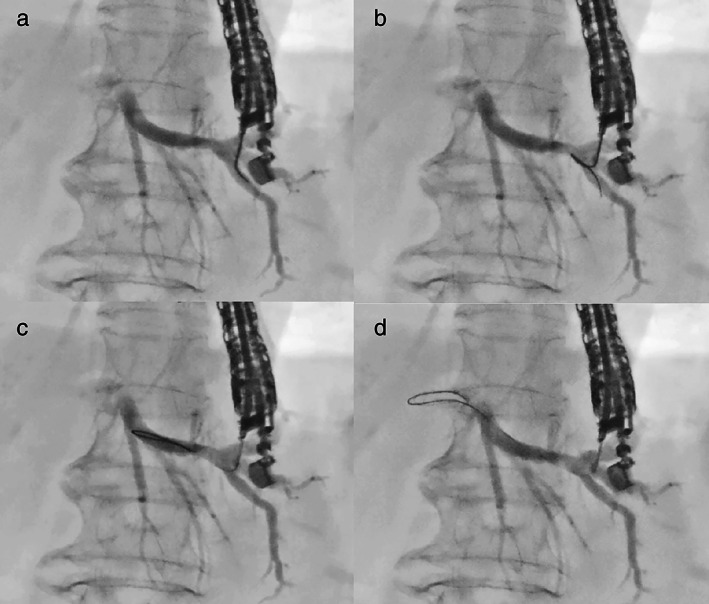
Endoscopic ultrasound‐guided hepaticogastrostomy with loop technique. (a) The guidewire is initially advanced toward the peripheral side. (b) The tip of guidewire is inverted. (c) Looped guidewire is advanced toward the hilar side. (d) The guidewire has successfully reached the left hepatic duct.

Guidewire manipulation is classified into two types based on the shape of the guidewire (Fig. 1). “Conventional technique” refers to selecting the correct direction by rotating the angled tip of the guidewire toward the hilum. In contrast, the “Loop technique” is when the guidewire tip is pressed against the wall of the bile duct or minor branches and inverted. The looped guidewire is then advanced into the larger bile duct using gentle pressure (Fig. 2). Initially, we attempted the conventional technique in all cases. The loop technique was used in cases in which the guidewire tip spontaneously inverted as a loop shape, or the guidewire advanced toward the peripheral side. The loop shape was formed by pushing the guidewire tip to the bile duct wall or minor branches. Until the guidewire was inserted sufficiently, the FNA needle was not replaced to the other device to prevent guidewire dislocation.

### Evaluation criteria from EUS‐HGS procedure

**Figure 3:**
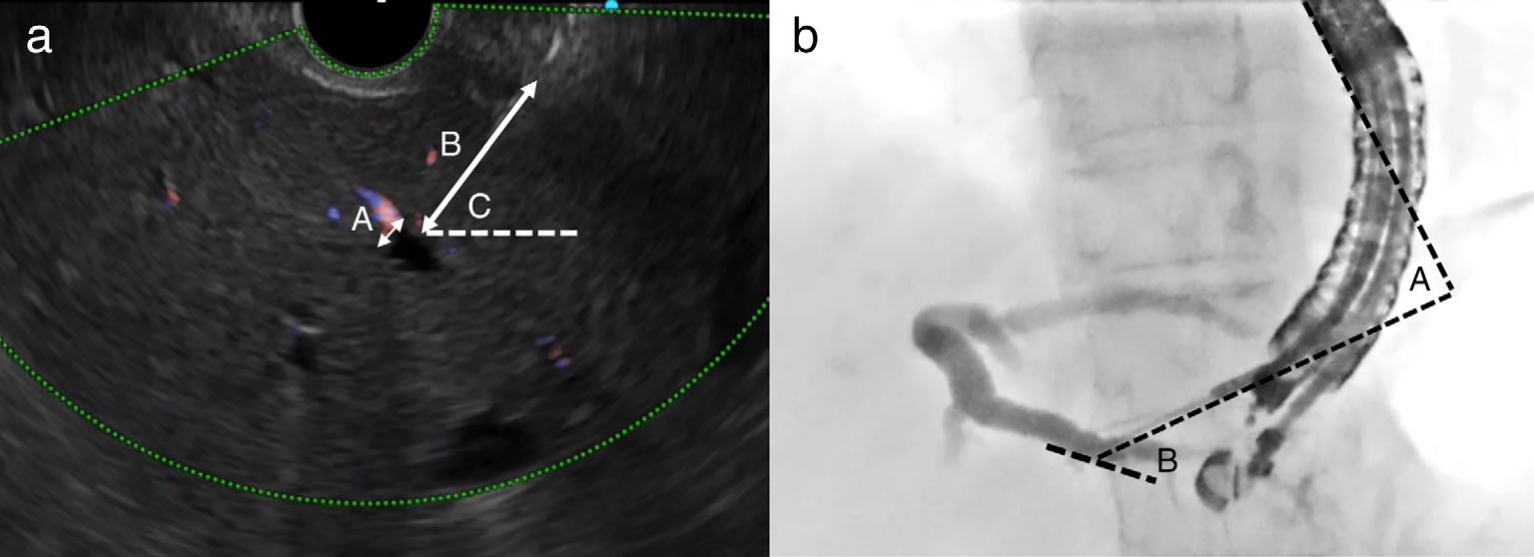
Evaluation criteria (a) (A) Diameter of punctured bile duct. (B) Length of punctured tract. (C) Angle of needle. (b) (A) Angle between the needle and the scope. (B) Angle between the bile duct and the needle.

All EUS and fluoroscopy movies and still images were retrospectively evaluated. The diameter of the punctured bile duct, length of the punctured tract, and angle of the needle were calculated from the still EUS images taken immediately before and after the puncture (Fig. 3). The shape of the punctured bile duct on EUS was classified into long or short axis. from the still fluoroscopic images taken immediately following injection of the contrast medium, two angles were calculated: the angle between the punctured bile duct and the needle, and the angle between the needle and the scope. Adobe Photoshop 2021 (Adobe Inc., California, USA) was used to measure the diameter, distance, and angle from the still images. The “initial direction of the guidewire”, that is, the direction in which the guidewire first advanced after it emerged from the needle (hilar or peripheral side), perforation of bile duct by the guidewire, and the type of guidewire manipulation (conventional or loop technique) were all noted based on the fluoroscopic movies. The chronological parameters with respect to the procedure were measured and defined as follows: “Guidewire insertion time” was the time it took for the guidewire to emerge from the tip of needle and enter the left hepatic duct. “Overall procedure time” was the time elapsed from the initial puncture to stent deployment. If multiple punctures were performed during a procedure, guidewire manipulation in the last session was analyzed. Adverse event associated with guidewire manipulation was defined as complete disconnection of the guidewire.

### Statistical analysis

All statistical analyses were performed using the JMP Pro version 15 (SAS Institute Inc., Cary, NC, USA). Technical success was defined as successful stent deployment, and successful guidewire manipulation was defined as the guidewire reaching the left hepatic duct. Continuous variables are presented as medians with interquartile ranges (IQR), and categorical variables are presented as frequency (*n*) and proportion (%). The cut‐off values and the area under the curves (AUC) of each angle were assessed using receiver operating characteristic (ROC) curves. Factors associated with the loop technique were assessed using the chi‐square test as well as Fisher's exact test for categorical variables, and Student's *t*‐test for continuous variables. Multivariable analysis was performed using logistic regression analysis. A *P‐*value <0.05 was considered statistically significant.

## Results

A total of 52 patients (34 male, and 18 female) with a median age of 73 years (IQR 69–80 years) were included in this study. The baseline characteristics of these patients and the details of their EUS‐HGS procedure are summarized in Table 1. The primary disease was malignant in 44 patients (85%) and benign in 8 (15%) of them. The indications for performing EUS‐HGS were duodenal obstruction (*n* = 27, 52%), hilar biliary obstruction (*n* = 13, 25%), surgically altered anatomy (*n* = 9, 17%), and difficult cannulation (*n* = 3, 5.8%). Ten patients (19%) had external drainage tubes placed prior to EUS‐HGS.

**Table 1:** Patient characteristics

Total number of patients, *n*	52
Median age (year), IQR	73, 69–80
Gender (male: female), *n* (%)	34:18, (65:35)
Disease, *n* (%)	
Pancreatic cancer	20 (38%)
Biliary tract cancer	12 (23%)
Gastrointestinal cancer	7 (13%)
Others	13 (26%)
Reason for EUS‐HGS, *n* (%)	
Duodenal obstruction	27 (52%)
Hilar biliary obstruction	13 (25%)
Altered anatomy	9 (17%)
Difficult cannulation	3 (5.8%)
External drainage, *n* (%)	10 (19%)
ENBD/PTBD	8 (15%)/2 (3.8%)
Technical success rate	100%
Number of punctures, *n* (%)	
1/2/3	43 (83%)/6 (12%)/3 (5.7%)
Punctured bile duct, *n* (%)	
B3/B2	41 (79%)/11 (21%)
Diameter of puncture site (mm) median, IQR	3.4 mm, 2.6–4.8 mm
>3 mm /≤3 mm, *n* (%)	34 (65%)/18 (35%)
Length of puncture path (mm) median, IQR	17, 14–22
Angle of needle on EUS (°) median, IQR	53, 50–57
Shape of bile duct on EUS, *n* (%)	
Long axis/short axis	23 (44%)/29 (56%)
Angle between bile duct and needle (°) median, IQR	122°, 28–88°
Angle between needle and endoscope (°) median, IQR	87.5°, 60–118°
Initial direction of GW, *n* (%)	
Hilar side/peripheral side	29 (56%)/23 (44%)
GW perforation of bile duct	8 (15%)
GW insertion method, *n* (%)	
Conventional technique/loop technique	32 (62%)/20 (38%)
GW insertion time (s) median, IQR	49, 20–123
Dilation method, *n* (%)	
Balloon dilator/mechanical dilator/electrocautery dilator	38 (73%)/12 (23%)/2 (3.8%)
Stent, *n* (%)	
Plastic stent/self‐expandable metallic stent	19 (37%)/33 (63%)
Procedure time (min) median, IQR	20.5, 17–30

ENBD, endoscopic nasal biliary drainage; EUS‐HGS, endoscopic ultrasound‐guided hepaticogastrostomy; GW, guidewire; IQR, interquartile range; PTBD, percutaneous transhepatic biliary drainage.

The number of punctures required for EUS‐HGS was 1 in 43 patients (83%), 2 in 6 patients (12%), and 3 in 3 patients (5.8%). Guidewire insertion into the left hepatic duct was successfully performed in all patients, and the technical success rate of the EUS‐HGS procedure was 100% (52/52). B3 was punctured in 41 patients (79%) and B2 in 11 patients (21%). The shape of the bile duct on EUS was along the long axis in 23 patients (44%) and along the short axis in 29 patients (56%). The initial direction of the guidewire was hilar in 29 patients (56%) and peripheral in 23 patients (44%). Successful guidewire manipulation was performed in 32 patients (62%) using the conventional technique and 20 patients (38%) using the loop technique. Guidewire perforation of the bile duct was observed in eight patients (15%); there was no significant difference between both techniques. The median guidewire insertion time was 49 s (IQR 20–123 s) and the overall procedure time was 20.5 min (IQR 17–30 min). No adverse events were associated with guidewire manipulation.

The cut‐off values of each angle were assessed using an ROC curve to check for the presence of any correlation with the use of the loop technique as follow: 54° for angle of needle on EUS (AUC: 0.555), 71°for angle between the bile duct and needle (AUC: 0.764), and 114°for angle between the needle and endoscope (AUC: 0.624). A univariate analysis of any correlation with the usage of the loop technique is presented in Table 2. Cases where the angle between the bile duct and the needle was >70° (*P* < 0.001) as well as cases wherein the initial direction of the guidewire was to the peripheral side (*P* < 0.001) were significantly more frequent in patients in whom the loop technique was used. The guidewire insertion time was significantly longer in patients in whom the loop technique was used (*P* = 0.012); however, there was no significant difference in the overall procedure time (*P* = 0.940). Logistic regression analysis revealed that an angle between the bile duct and needle >70° was independently associated with the use of the loop technique (odds ratio (OR): 9.84; 95% confidence interval [CI]: 2.24–43.13; *P* < 0.01) (Table 3).

**Table 2:** Univariate analysis associated with loop technique

	Conventional technique (*N* = 32)	Loop technique (*N* = 20)	*P*‐value
Age (years) median (IQR)	74 (70–81)	72.5 (66–78)	0.127^†^
Age >75 years	11 (34%)	6 (30%)	1.000^†^
Gender (male)	23 (72%)	11 (55%)	0.244^†^
Malignant	27 (84%)	17 (85%)	1.000^†^
Duodenal obstruction	17 (53%)	10 (50%)	1.000^†^
Altered anatomy	7 (24%)	2 (10%)	0.285^†^
Puncture site (B3)	26 (81%)	15 (75%)	0.730^†^
External drainage	7 (22%)	3 (15%)	0.723^†^
Diameter of puncture site (mm) median (IQR)	3.5 (2.7–4.7)	3.4 (2.3–4.8)	0.853*
Diameter of puncture site >3 mm	23 (72%)	11 (55%)	0.244^†^
Length of puncture path (mm) median (IQR)	17 (14–22)	19 (14–25)	0.64*
Length of puncture path >2 cm	9 (28%)	10 (50%)	0.144^†^
Angle of needle on EUS (°) median (IQR)	52 (49–57)	54.5 (50–57)	0.625*
Angle of needle on EUS <55°	13 (41%)	12 (60%)	0.255^†^
Axis of bile duct on EUS (long axis)	13 (41%)	10 (50%)	0.5736^†^
Angle between bile duct and needle (°) median (IQR)	44.5 (15.5–64.5)	87 (60.5–108)	<0.001*
Angle between bile duct and needle >70°	5 (16%)	15 (75%)	<0.001^†^
Angle between needle and endoscope (°) median (IQR)	79 (56–109)	100.5 (66–129)	0.131*
Angle between needle and endoscope >110°	7 (22%)	9 (45%)	0.123^†^
Initial direction of GW (peripheral side)	8 (25%)	15 (75%)	<0.001^†^
GW perforation of bile duct	3 (9.4%)	5 (25%)	0.235
GW insertion time (s) median (IQR)	39.5 (15–93)	68 (33–264)	0.012*
GW insertion time >60 s	12 (38%)	10 (50%)	0.403^†^
Procedure time (s) median (IQR)	20 (18–30)	26.5 (16–34)	0.940*
Procedure time >20 min	14 (44%)	12 (60%)	0.393^†^

* Fisher's exact test.

^†^ Student *t*‐test.

GW, guidewire; IQR, interquartile range.

**Table 3:** Logistic regression analysis of loop technique

	OR	95% CI	*P*‐value
Angle between bile duct and needle >70°	9.84	2.24–43.13	<0.01
Initial direction of GW (peripheral side)	4.36	0.99–19.13	0.051

OR, odds ratio; CI, confidence interval; GW, guidewire.

## Discussion

EUS‐guided biliary drainage is mainly classified into two techniques depending on the drainage route: choledocoduodenostomy (CDS) and HGS.^4^, ^5^ EUS‐HGS has the advantage of being viable in surgically altered anatomy and hilar obstructions.^18^ In a randomized multicentric trial, Minaga *et al*. reported that HGS was not inferior to CDS in terms of technical success (HGS: 87.5%, CDS: 82.6%; *P* = 0.028) and early adverse events (HGS: 8.3%, CDS: 8.7%; *P* = 0.965).^19^ Artifon *et al*. also reported similar outcomes when comparing HGS and CDS with respect to technical success rates (HGS: 96%, CDS: 91%) and adverse events (HGS: 20%, CDS: 12.5%).^20^ However, a systematic review by Khan *et al*. reported that CDS appeared to be significantly safer than HGS (pooled OR: 0.40; 95% CI: 0.18–0.87).^3^

Several techniques and devices have been developed to increase the success rate and reduce the adverse events associated with EUS‐HGS. To puncture the non‐dilated bile duct, a 22‐G FNA needle with a 0.018‐in. guidewire is preferred.^8^, ^21^ Although tract dilation is one of the most difficult steps of EUS‐HGS, the novel mechanical or balloon dilator has made it easier and safer.^22^, ^23^, ^24^ With regard to stent deployment, novel metallic stents with thin delivery systems have been developed to facilitate their insertion into the bile duct.^13^ A dedicated plastic stent is also available.^14^ The intra‐scope channel stent release technique has been reported to prevent stent migration into the abdominal cavity.^12^ Despite these advancements in techniques and devices, there are still no dedicated guidewires or effective guidewire manipulations for EUS‐HGS. Vila *et al*. reported a lower success rate and higher incidence of adverse events for EUS‐guided transluminal drainage in trainees, which was attributed to guidewire manipulation issues in 68.2% of cases.^25^ Therefore, it is necessary to develop safer and more reliable guidewire manipulation techniques for use in EUS‐HGS.

Regarding guidewire manipulation in EUS‐HGS, it is important to determine in which direction the guidewire advances just after it emerges from the needle. If the guidewire initially advances toward the hilar side, subsequent manipulation is relatively easy, requiring only rotation of the angled tip. However, if the guidewire is emerging toward the peripheral side, the tip of the guidewire must be retracted and rotated toward the hilar side; this manipulation often results in guidewire shearing. To prevent this, we used a liver impaction technique that pulled the FNA needle into the liver parenchyma.^9^ However, once the tip of the FNA needle is pulled, there is a risk that the guidewire will be pulled out of the bile duct in the same direction, making reinsertion impossible. To overcome this difficulty, novel guidewire manipulation techniques must be developed.

A few reports have demonstrated guidewire manipulation techniques used during the course of EUS‐HGS. Ogura *et al*. reported the effect of the echoendoscope angle on the success of guidewire manipulation.^15^ In that report, the cut‐off value of the angle between the needle and the scope was determined to be under 135° as per the ROC curve, and it was a statistically significant factor linked to successful guidewire manipulation (OR: 0.03; 95% CI: 0.01–0.04; *P* < 0.05). To create this angle, one must raise both the up‐angle and elevator of the endoscope simultaneously. However, in practice, it is necessary to puncture while avoiding vessels; therefore, it is not always possible to create such an acute angle. Additionally, in this report, “successful guidewire manipulation” is defined as the initial emergence of the guidewire toward the hilar side of the bile duct. In our report, the guidewires initially advanced into the peripheral side in 44% (23/52) of patients, which was considered “unsuccessful guidewire manipulation” according to the above definition; however, technical success was achieved in all cases using the loop technique.

**Figure 4:**
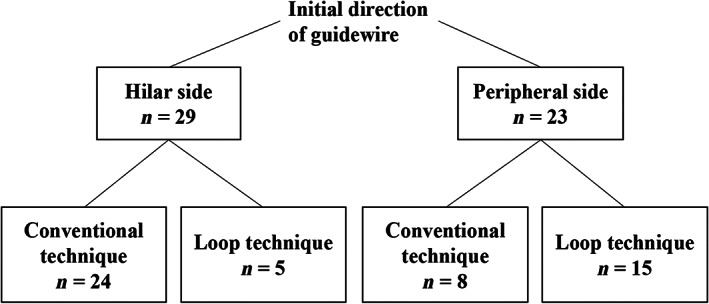
Details of guidewire manipulation.

The loop technique has advantages in difficult cases for guidewire manipulation during EUS‐HGS. First, the loop shape advances into the main branch toward the hilar side while preventing insertion into the minor branches. Second, even if the loop shape is made on the peripheral side, the tip of the guidewire can be turned toward the hilar side by carefully pulling the guidewire. In our case, the loop technique was used in 17% (5/29) of the patients in whom the guidewire initially advanced toward the hilar side and in 65% (15/23) patients in whom the guidewire initially advanced toward the peripheral side (Fig. 4).

Univariate analysis revealed two factors that were significantly correlated with the loop technique: angle between the bile duct and the needle of >70° (*P* < 0.001), and initial guidewire emergence toward the peripheral side (*P* < 0.001). Oh *et al*. reported that the diameter of the bile duct (>5 mm) and the length between the bile duct and the mural wall (≤3 cm) were risk factors for technical failure in EUS‐HGS.^26^ In our study, these factors were not significantly correlated with the use of the loop technique. Logistic regression analysis showed that only the angle between the bile duct and needle >70° is independently correlated with the use of the loop.

Our study revealed the conditions under which the loop technique was used for guidewire manipulation during EUS‐HGS. Among patients with an angle between the bile duct and needle was >70°, the loop technique was used in 75% (15/20) of patients, whereas 16% (5/32) of patients had the conventional techniques used in their procedure. This result indicates that we can use the information from fluoroscopic images to decide on subsequent guidewire manipulation. In addition, the loop technique is a safe procedure, as safe as conventional technique, since there were no adverse events recorded during guidewire manipulation.

This study had several limitations. First, since this was a retrospective study, the two types of guidewire manipulation were not selected for use in a patient based on any pre‐existing criteria. Therefore, it is necessary to conduct a prospective study with a larger sample size. Second, this study was limited to patients in whom a 19‐G FNA needle and a specific 0.025‐in. guidewire were used. Although this criterion was determined to reduce selection bias, the ability to form a loop may be different in other guidewires.^27^ Furthermore, the efficacy of the recently reported 0.018‐in. guidewire should be assessed in the future.^8^, ^21^

## Conclusion

In conclusion, our study, for the first time, established the utility of the loop technique in EUS‐HGS and the optimal conditions for its use. This technique is recommended if the angle between the bile duct and needle is >70° on fluoroscopic images.

## Data Availability

The datasets used and analyzed in the current study are available from the corresponding author on reasonable request.
